# Mechanisms of resistance to FGFR1 inhibitors in FGFR1-driven leukemias and lymphomas: implications for optimized treatment

**DOI:** 10.20517/cdr.2021.30

**Published:** 2021-05-25

**Authors:** John K. Cowell, Tianxiang Hu

**Affiliations:** Georgia Cancer Center, 1410 Laney Walker Blvd, Augusta, GA 30912, USA.

**Keywords:** MLN-eo FGFR1, lymphomas, FGFR1, mutations, PTEN, PUMA, mouse models

## Abstract

Myeloid and lymphoid neoplasms with eosinophilia and FGFR1 rearrangements (MLN-eo FGFR1) disease is derived from a pluripotent hematopoietic stem cell and has a complex presentation with a myeloproliferative disorder with or without eosinophilia and frequently presents with mixed lineage T- or B-lymphomas. The myeloproliferative disease frequently progresses to AML and lymphoid neoplasms can develop into acute lymphomas. No matter the cell type involved, or clinical presentation, chromosome translocations involving the *FGFR1* kinase and various partner genes, which leads to constitutive activation of downstream oncogenic signaling cascades. These patients are not responsive to treatment regimens developed for other acute leukemias and survival is poor. Recent development of specific FGFR1 inhibitors has suggested an alternative therapeutic approach but resistance is likely to evolve over time. Mouse models of this disease syndrome have been developed and are being used for preclinical evaluation of FGFR1 inhibitors. Cell lines from these models have now been developed and have been used to investigate the mechanisms of resistance that might be expected in clinical cases. So far, a *V561M* mutation in the kinases domain and deletion of *PTEN* have been recognized as leading to resistance and both operate through the PI3K/AKT signaling axis. One of the important consequences is the suppression of PUMA, a potent enforcer of apoptosis, which operates through BCL2. Targeting BCL2 in the resistant cells leads to suppression of leukemia development in mouse models, which potentially provides an opportunity to treat patients that become resistant to FGFR1 inhibitors. In addition, elucidation of molecular mechanisms underlying FGFR1-driven leukemias and lymphomas also provides new targets for combined treatment as another option to bypass the FGFR1 inhibitor resistance and improve patient outcome.

## INTRODUCTION

**Figure 1.**
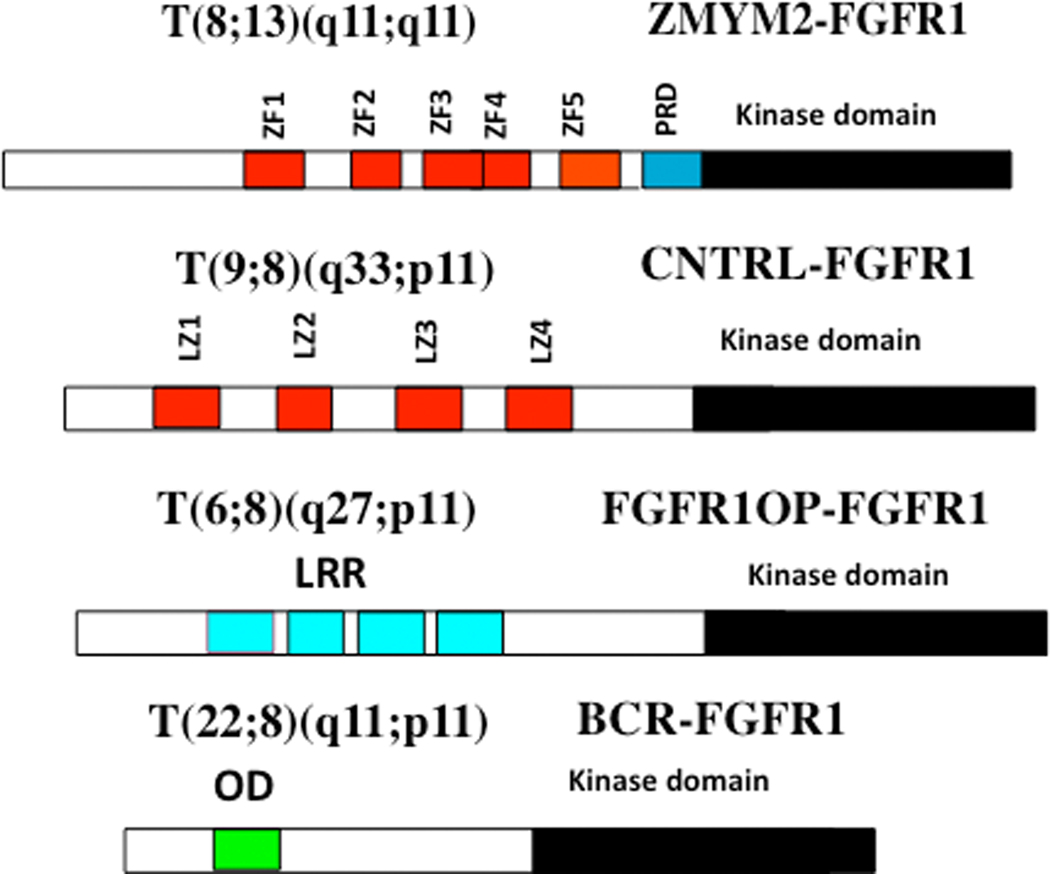
Examples of chimeric kinase motifs provided by partner genes include Zinc Fingers (ZF), Leucine zippers (LZ), Leucein rich repeats (LRR) and oligomerization domains (OD) all of which facilitate dimerization.

**Figure 2.**
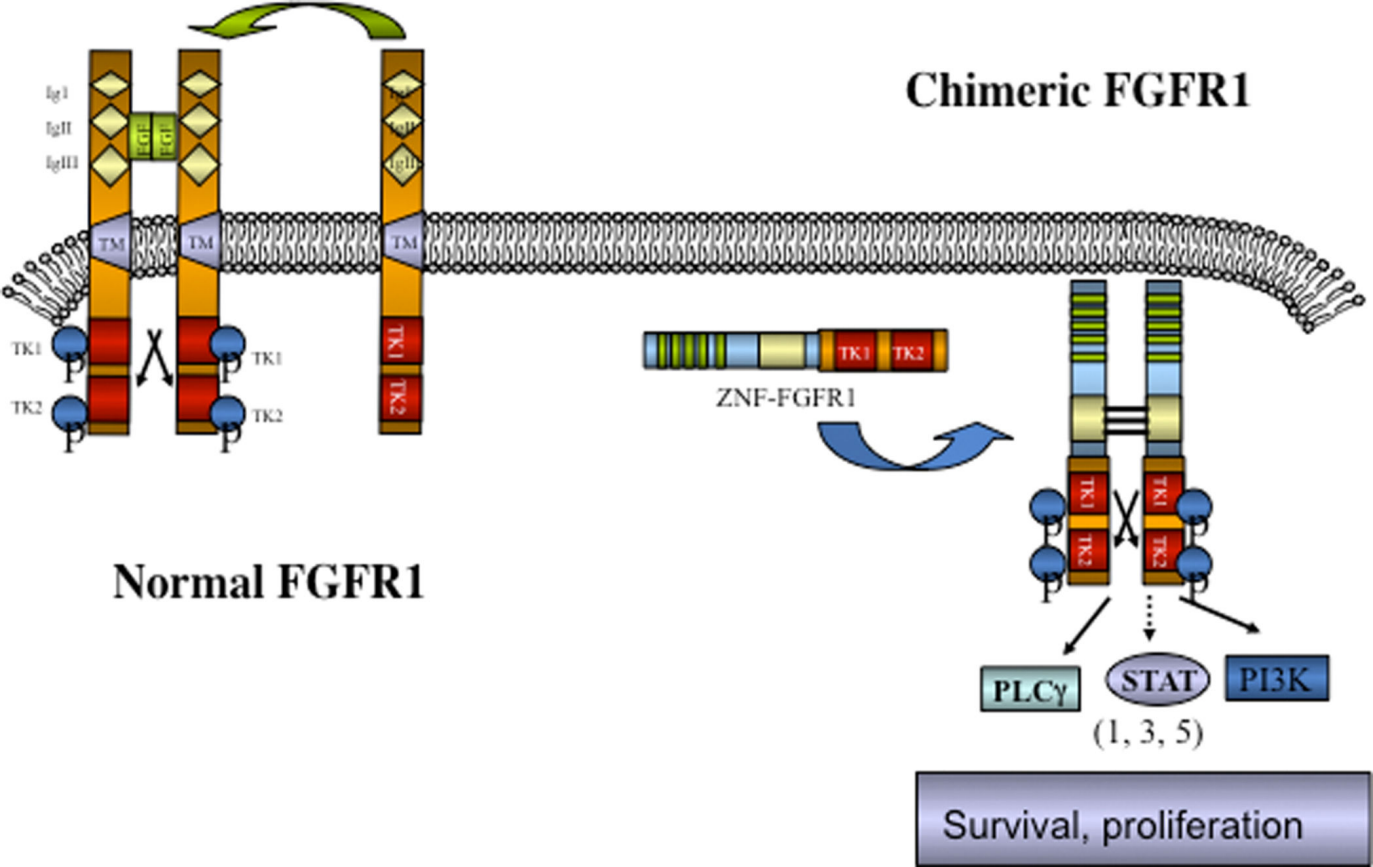
Schematic of the activation of chimeric kinases. Normal monomeric, membrane bound, FGFR1, on binding ligand, dimerizes and is phosphoactivated but remains tethered to the membrane. In the absence of ligand FGFR1 remains an inactive monomer. Chimeric kinases are dimerized through the partner gene motif leading to ligand independent activation and constitutive downstream signaling.

The involvement of chromosome region 8p11 in an atypical myeloproliferative disease was first described in 1992^[1]^ and subsequently referred to^[2,3]^ as 8p11 myeloproliferative syndrome (EMS). This syndrome is characterized cytogenetically by the presence of reciprocal chromosome translocations that invariably involve 8p11, often as the only cytogenetic abnormality^[4]^, and is the only example of a malignancy that always involves abnormalities of *FGFR1*. At least 15 different chromosome translocations have been associated with this syndrome^[5–7]^ but the invariant features are the translocation breakpoints occurring in intron 8 of FGFR1, just upstream of the kinase domain, and the presence of a dimerization/oligomerization domain in the partner translocated gene [Figure 1]. The most common rearrangements are t(8;13)(p11;q12), t(8;22)(p11;q11) and t(8;9)(p12;q33) giving rise to ZMYM2-FGFR1, BCR-FGFR1 and CNTRL-FGFR1 fusion kinases respectively. As a result of the rearrangement, an in-frame fusion event occurs in all cases, facilitating the activation of FGFR1 kinase as a result of dimerization and, since the partner genes are typically constitutionally active so too is the fusion kinase. The loss of the trans-membrane domain from *FGFR1* in the fusion genes leads to constitutive and ligand-independent activation, which is responsible for the transformation of hematopoietic stem cells [Figure 2]. These rearrangements give rise to mixed lineage disease^[8]^ suggesting a pluripotent stem cell origin, which led to a nomenclature of the Stem Cell Leukemia/Lymphoma (MLN-EO FGFR1) syndrome^[9]^. This syndrome has since been classified by the WHO^[10]^ as myeloid and lymphoid malignancies associated with eosinophilia and FGFR1 rearrangement (MLN-eo FGFR1). Because of phenotypic overlap in clinical presentation, rearrangements involving *PDGFR*A and *PDGFRB* were added to the sub-classification^[10,11]^, although there are still important clinical differences between each subgroup^[6]^.

## CLINICAL ANALYSIS

### Presentation characteristics

The diagnosis of MLN-eo FGFR1 is complicated by the mixed lineage T-, B- and myeloid cell immunephenotypes on the malignant cells suggesting a pluripotential stem cell origin. Patients show peripheral blood leukocytosis and splenomegaly due to extra medullary hematopoiesis. Typically presentation is of a chronic myeloproliferative neoplasm (MPN) with variable presence of eosinophilia^[10]^. These patients may also transform to acute lymphoblastic leukemia and/or AML within 1–2 years while some patients present with AML without the antecedent MPN. Depending on the specific chromosome translocation involved, these patients may also demonstrate T-lymphoblastic lymphoma, with aggressive development of blast phase secondary acute leukemia with a myeloid phenotype^[9]^ or, as in the case of patients with the BCR-FGFR1 t(8;22) rearrangement, B-ALL and peripheral basophilia^[12–15]^. Cytogenetic analysis shows that the same fusion gene is present in the myeloid or lymphoid malignancies of the patient, regardless of their cellular immune phenotype, suggesting a common underlying cause^[16,17]^. A detailed summary of the variable clinical characteristics associated with MLN-eo FGFR1 patients have been described in detail elsewhere^[4,9,14,18]^.

The t(8;13p)(11q12) rearrangement is the most common structural chromosome translocation which was shown to generate the *ZNF198* (*ZMYM2*)*-FGFR1* chimeric gene^[19]^ that typically leads to T-lymphomas^[9]^. Other, less common, rearrangements that also lead to T-lymphomas include *FGFR1OP2-FGFR1*^[20]^ and *CNTRL-FGFR1*^[21,22]^. Rearrangements involving the *BCR* gene, however, are more frequently associated with B-lymphomas^[12,14,23]^. While there has been extensive characterization of the function of the chimeric kinases *in vitro*^[24–26]^, the common observation is the involvement of constitutive activation of FGFR1 leading to downstream activation of a variety of signaling pathways, although it has been proposed that the fusion partner can possibly influence the phenotypic outcome and survival characteristics from studies in animal models^[27]^.

While the activation of FGFR1 through chromosome translocations is the hallmark of MLN-eo FGFR1, and considered the driver event in transfomation of HSCs, it has also been reported that additional cytogenetic abnormalities can occur in the leukemic cells, particularly associated with progression to blast phase^[4–5,14]^. In particular, trisomy 21 was seen as the most common secondary abnormality associated with progression^[5,28]^. There have also been reports of mutations involving *RUNX1* frequently accompanying *FGFR1* rearrangements^[28–30]^ during progression. Thus, it is possible that these additional genetic changes may influence not only progression but also the phenotype of the leukemic cells and may possibly modify the response to FGFR1 inhibitors.

### Incidence of MLN-eo FGFR1

MLN-Eo FGFR1 is generally described as an exceeding rare syndrome, where the incidence has been hard to quantify. The complex diversity in its presentation characteristics may contribute to under diagnosis and, unless definitive cytogenetic or molecular analysis is performed, may go undiagnosed. In a study from the Mayo Clinic involving over 24,000 sequential cases of leukemia analysed cytogenetically from this institution, only 4 cases (0.000164%) showed an FGFR1 rearrangement^[4]^. This, on a background of leukemias that represent only ∼10% of all cancers confirms the rare nature of the disease in the population. As a result, our understanding of its pathobiology has been limited and conducting clinical trials has been challenging. Consequently, the majority of case reports describe only a single patient with limited molecular analysis and, where drug treatments were reported only single cases were involved^[7,22,31–33]^. To overcome these limitations, several groups have developed murine models of the disease to study the genetic changes responsible for transformation of the hematopoietic stem cell and to investigate the use of molecularly targeted therapies in suppressing the disease without the limitations of incidence and variable diagnosis imposed in human populations.

## EXPERIMENTAL STUDIES OF DRUG RESISTANCE

### Mouse models of MLN-eo FGFR1

Early models of *MLN-eo FGFR1*^[34,35]^ involved transduction of hematopoietic stem cells with either the *ZMYM2-FGFR1* or *BCR-FGFR1* chimeric kinases. When transplanted into syngeneic hosts, the transformed bone marrow cells give rise to MLN-eo FGFR1 disease in the recipient mice, which faithfully recapitulated the splenomegaly and peripheral leukocytosis seen in the human disease. These models also developed MPN and associated lymphomas typical of the human disease. Similar models were developed independently and showed comparable phenotypes and disease progression^[36–38]^. In the *ZMYM2-FGFR1* model reported by Ren *et al*.^[39]^, the development of T-lymphomas highlighted their close genetic relationship with T-lymphomas from other origins, notably the upregulation of the Notch signaling pathway and the role of BCL2^[39]^. Subsequently other mouse models were developed for the BCR-FGFR1^[37]^, CNTRL-FGFR1^[38]^ and FGFR1OP2-FGFR1^[40]^ chimeric kinases where, for the most part, the disease developed with a similar presentation to the human disease, although transformation of the myeloproliferative disorder to AML only occurred in the *CNTRL-FGFR1* and *FGFR1OP2-FGFR1* models. We have argued in the other cases the rapid onset of lymphomas may have overwhelmed the mice, which died before transformation to AML could occur. To extend these studies to be more representative of the human disease, *in vivo* models have also been developed for human cells derived from CD34+ cord blood cells transformed with the chimeric kinases. Using the same transduction and transplantation approaches developed for the mouse models, transplantable BCR-FGFR1^[41]^, CNTRL-FGFR1^[38]^ and ZMYM2-FGFR1 transformed human cells^[42]^ were xenografted into immune-compromised mice where disease progression and presentation closely mimicked development of MPN and AML in the human disease after relatively long latency periods.

Cell lines were developed from the mouse models which, when xenografted into mice also produced representative MLN-eo FGFR1 disease. BBC2 cells express BCR-FGFR1 and lead to rapid (< 20 days) onset of B-cell leukemia/lymphoma. Both the *ZMYM2-FGFR1* expressing ZNF112 and *CNTRL-FGFR1* expressing CEP2A cells predominantly show a T-cell ALL immunophenotype. The *FGFR1OP2-FGFR1* chimeric kinase was also detected^[43]^ in a human cell line, KG1, which had unwittingly been developed 30 years earlier in Mel Greaves’s laboratory^[44]^. These cell lines have been used to develop concepts for drug resistance.

### Treatment of MLN-eo FGFR1.

During the early years following recognition of MLN-eo FGFR1, treatments used were largely adapted from standard regimens in place for ALL or AML^[9,18]^, where prognosis was typically poor. Clinical course of the disease is very aggressive with rapid progression to secondary acute leukemia within 1–2 years. In one early study of 65 cases, clinical remission rate was 27% and overall survival was 15 months after intensive chemotherapy^[9]^. Hematopoietic stem cell transplants (HSCT) were clearly indicated to improve these statistics but in the same study, mean survival time for transplant patients was only increased to 24 months. In a more recent study, although prognosis remained poor, allogenic HSCT provided some clinical benefit in selected patients^[15,18]^ and provides the only possibility of curative treatment. Using multi-targeted kinase inhibitors such as PKC412 (midostaurin) and sorafinib only provided a short term hematological response^[34,45]^. The next generation of more specific drugs, largely only targeting the FGFR family, included ponatinib, which was originally developed to target the mutant BCR-ABL rearrangement in CML^[46]^ but showed a reasonable specificity for FGFR1 at lower concentrations. In one case, after showing chemotherapy resistance, partial remission was achieved using ponatinib^[32]^, although other studies suggested it was less effective^[6]^. Recently more successful remission was achieved using pemigatinib on a single patient with a t(8;9) rearrangement, although the patient died of unrelated complications^[7]^.

### Development of FGFR1 inhibitor regimens in mouse models of MLN-eo FGFR1

There have been suggestions about potential molecular targets in MLN-eo FGFR1 based on genetic studies in the mouse models of *MLN-eo FGFR1* (see below). However, since the most consistent observation is the constitutive activation of FGFR1, this seemed like the rational target that might treat all manifestations of the disease whether T-, B-, stem or myeloid cells are involved, since they all carry the same FGFR1 kinase component. The mouse models described above have been used to assess the efficacy of using FGFR1 inhibitors that may translate to the human disease. In an extensive survey of the effects of ponatinib in a variety of cells expressing different fusion kinases, for example, we reported a remarkable efficacy in suppressing leukemogenesis in cell line xenografts in mice^[47]^. Since these early observations, however, several more specific inhibitors have been developed including the BGJ398^[48]^, JNJ 42756493^[49]^ and AZT4548^[50]^. In a side-by-side comparison of the effectiveness of these drugs in suppressing leukemogenesis *in vitro*, we demonstrated efficient suppression of cell growth, with BGJ398 being the most effective and at micro molar concentrations as well as improving survival in mouse models *in vivo*^[51]^. The exciting use of mouse models to investigate the potential utility of specific drugs to treat MLN-eo FGFR1 led to our studies that investigated the mechanisms of resistance to FGFR1 inhibitors.

### Genetics of resistance to FGFR1 inhibitors

In anticipation of the more widespread use of FGFR1 targeted therapies for MLN-eo FGFR1 in the future, we investigated possible mechanisms of resistance to several FGFR1 inhibitors in the murine models. For the most part, the mode of action of FGFR1 inhibitors is to target the ATP binding domain of the FGFR1 kinases and so share a common mechanism with greater or lesser effectiveness. Various T- and B-lymphoma murine cell lines derived from primary tumors expressing *ZMYM2-FGFR1*^[36]^, *CNTRL-FGFR1*^[38]^ and *BCR-FGFR1*^[37]^, as well as the human KG1 cell line, which expresses the *FGFR1OP2-FGFR1* kinase, were treated with progressively increasing concentrations of ponatinib over many months leading to populations that became 1000–3000-fold resistant^[52]^. As anticipated from the underlying targeting mechanisms, cells that were resistant to ponatinib were also resistant to other inhibitors such as BGJ398, AZT4548 and JNJ42756493.

Consistent with targeting of the ATP binding domain, most mechanisms of resistance to tyrosine kinase inhibitors result from mutations in the ATP binding site^[53]^. Sequencing the FGFR1 kinase domain in the chimeric genes in the four resistant cell lines showed that two of them carried the same *V561M* mutation in the ATP binding domain. Chemical biology studies using bacterial synthesized proteins *in vitro* demonstrated that the mutant site was a significant activating event and that E3810^[54]^ and AZT4547 showed a reduced binding affinity to the mutant protein^[55]^ and induced strong resistance to PD173074 and BGJ398. In model cell systems, the *V561M* mutation increased FGFR1 auto-phosphorylation leading to hyperactivation. Together these studies provide confirmatory evidence that the *V561M* mutation is responsible for resistance to FGFR1 inhibitors.

To assess the mechanism in the other 50% of the cell lines, we investigated changes in cancer cell related signaling pathways using the representative proportional protein analysis. This technology uses antibodies not only to common cancer dysregulated genes but also their target phosphorylation sites in an array format to report changes in the resistant *vs*. sensitive cell pairs. Resistant MLN-EO FGFR1 cells that did not show the *V561* mutation, instead showed deletion of exon 6 in the *PTEN* gene in both cases leading to a premature stop codon that abolishes PTEN function. Introduction of a wild type *PTEN* gene into these resistant cells re-conferred sensitivity to the FGFR1 inhibitors^[52]^. PTEN suppresses PI3K signaling^[56]^ and, as a result of its deletion, the PI3K/AKT pathway was upregulated in the resistant cells^[52]^. The significance of PTEN upregulation in the resistant cells was re-enforced following pharmacological inhibition of PI3K activity using BEZ235, which led to reduced proliferation *in vitro* and increased survival *in vivo*^[52]^.

### Mechanisms underlying resistance to FGFR1 inhibitors

To extend our understanding of the downstream consequences of the mutations leading to resistance, we used RNA-Seq to compare gene expression changes in sensitive and resistant murine and human cell lines and demonstrated a consistent dysregulation of genes involved in various cell death pathways^[57]^. Particularly notable was the up regulation of the *Bbc3* gene, which encodes the p53 upregulated modulator of apoptosis (PUMA) protein, a potent killer from the BCL2 family of proteins^[58]^, which exerts its influence both through p53-dependent and independent mechanisms^[59]^. The proapoptotic PUMA protein regulates apoptosis by interacting with antiapoptotic members of the BCL2 family of proteins. As a result, the proapoptotic proteins BAX and BAK are released from the mitochondrial membrane resulting in activation of their proapoptotic action. In the absence of PUMA, BCL2 retains its strong suppressive influence over apoptosis^[58]^.

**Figure 3.**
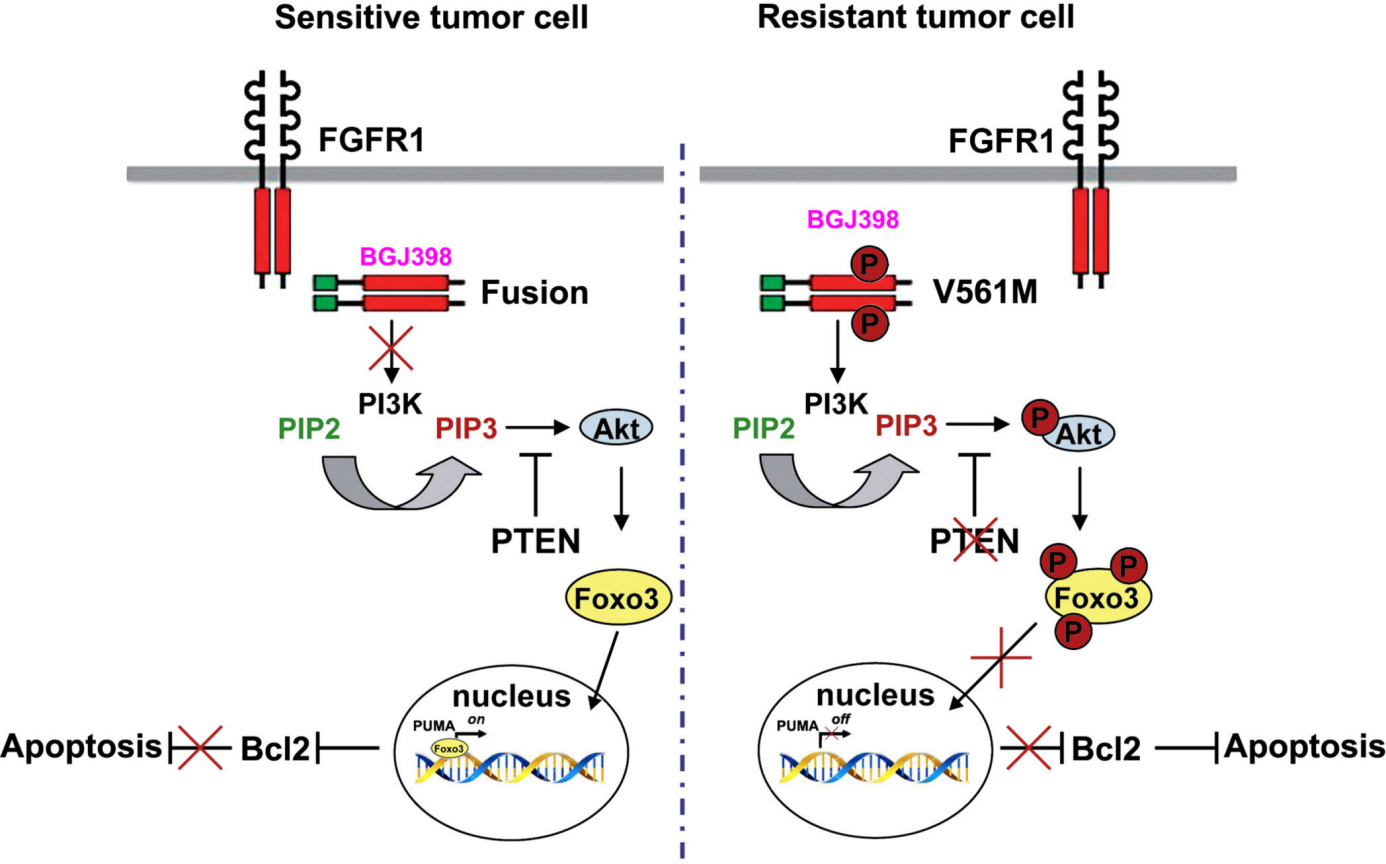
Mechanisms of resistance to FGFR1 inhibitors. In sensitive cells, treatment with an inhibitor (e.g., BGJ398) prevents activation of PI3K. As a result AKT is not activated and FOXO3A remains unphosphorylated and can move into the nucleus where it activates PUMA. PUMA then sequesters the antiapoptotic *BCL2* gene, removing its restraint on apoptosis. In the resistant cells, BGJ398 cannot bind FGFR1 and so PI3K is activated leading to activation of AKT and phosphorylation of FOXO3A which is sequestered in the cytoplasm. As a result, PUMA is not activated and so cannot interact with and suppress BCL2 and so apoptosis is suppressed.

In MLN-Eo FGFR1 cells, constitutive FGFR1 activation leads to activation of the PI3 Kinase, which in turn activates AKT [Figure 3] leading to downstream signaling cascades that promote oncogenesis. The *V561M* mutation in resistant cells is not affected by FGFR1 inhibitors and so AKT signaling is sustained. PUMA is regulated mostly at the transcription level and a number of different transcription factors have been implicated, including the 3A member of the forkhead (FOXO3A) transcription factor family^[60,61]^. AKT phosphorylation of FOXO3A sequesters it in the cytoplasm and so low levels of PUMA are maintained. Pharmacological suppression of FGFR1 activation, however, suppresses AKT activation by PI3K, allowing unphosphorylated FOXO3A to relocate into the nucleus and activate PUMA, which orchestrates apoptosis through BCL2. Since both *FGFR1* mutation and loss of PTEN impact the activation of AKT, FOXO3A is phosphoactivated in resistant MLN-Eo FGFR1 cells, thus suppressing PUMA activation.

Since BCL2 is a major response indicator of FGFR1 inhibition, when MLN-eo FGFR1 cells with either *FGFR1* mutations or PTEN deletions were treated with the potent ABT199 (ventocalx) BCL2 inhibitor^[62,63]^, there was a dose-dependent relative decrease in cell survival and reduced leukemic cell expansion *in vivo*^[57]^. Thus, targeting the PI3K pathway downstream of PUMA increases cell apoptosis and suppresses cell viability and may suggest an alternative therapeutic approach to target cells resistant to FGFR1 inhibitors.

BIM is another protein in the BCL2 family that has overlapping function with PUMA in promoting apoptosis by binding with BAK and BAX to destabilize the mitochondrial membrane^[64–67]^. In MLN-Eo FGFR1 cells resistant to FGFR1 inhibitors, BIM was also inactivated^[52]^. While BIM can be regulated by posttranslational modification, it can also be regulated at the transcription level where FOXO3A is the key transcriptional regulator^[68]^. Thus, deregulation of AKT activation in cells resistant to FGFR1 inhibitors affects two key regulators of apoptosis.

### An unsuspected mechanism to avoid sensitivity to FGFR1 inhibitors

Dimerization of the chimeric kinases in MLN-eo FGFR1 results in phospho-activation of FGFR1 and targeting the ATP binding site in the kinase has become the prime mechanism of suppressing FGFR1 activation, which is essential for transformation^[27]^. Activation of FGFR1 is presumed to phosphoactivate oncogenic downstream protein targets. Recently, however, it was shown that the chimeric kinases can be cleaved by granzyme B, which generates a truncated *FGFR1* (*nFGFR1*) derivative which localizes exclusively to the nucleus^[69]^. These nFGFR1 derivatives lack the dimerization domains provided by the translocated partner genes and, as such, are not dimerized and not phosphoactivated. Through a study of MYC activation in MLN-eo FGFR1 cells, nFGFR1 has been shown to function as a cofactor for transcription regulation^[69]^ and likely promotes expression of many other genes, some of which may be involved in transformation of HSCs. Since nFGFR1 does not have inherent DNA binding motifs, its function as a transcription regulator most likely involves associated with as yet unidentified partners. The critical role of granzyme B in generating the truncated nFGFR1 was shown through mutating the binding site and using pharmacological inhibitors. Treatment of BaF3 cells that express the fusion kinases with FGFR1 inhibitors *in vitro* shows suppression of cell growth but the cells transformed with nFGFR1 are insensitive to FGFR1 inhibitors. In serial transplant studies in mice, the nFGFR1 derivative became a dominant molecular species in advanced stages of disease evolution and indeed, in unpublished studies, we have shown that nFGFR1 can independently transform murine HSCs in *in vivo* transformation studies, albeit with a relatively long latency period. These observations have profound implications for the application of FGFR1 inhibitors as a means for treating MLN-eo FGFR1 since, although the full length kinases can be suppressed, the truncated derivative is still promoting mechanisms of oncogenesis that are not sensitive to these inhibitors. A better understanding of the molecular consequences of the generation of nFGFR1, therefore, will be essential to overcome this alternative mechanism of transformation.

### Alternative regimens to overcome FGFR1 resistance in MLN-eo FGFR1

Resistance to FGFR1 inhibitors in FGFR1 overexpressing malignancies will undoubtedly be a consequence of monotherapies, requiring alternative and/or combinatorial regimens to treat MLN-eo FGFR1. Of note, FGFR1 overexpression is not restricted to MLN-eo FGFR1 and has been shown in 10%−20% of *de novo* AML^[51]^, which are also sensitive to FGFR1 inhibition *in vivo*, thus broadening the relevance of overcoming resistance to FGFR1 inhibitors. Although new FGFR1 inhibitors will undoubtedly be developed, if targeting the ATP binding domain remains the main strategy, they will likely fall short of avoiding resistance too. As such, alternative approaches to overcome B- and T- lymphomas have been suggested from model systems.

Genomic analysis of T-lymphomas expressing chimeric FGFR1 kinases, for example, demonstrated deletion of the T-cell receptor alpha (*TCRA*) gene, upregulation of BCL2^[39]^ and increased expression of Notch^[39]^. Activation of notch requires cleavage by gamma secretase and, as an early proof-of-principle, treatment of these cells with gamma secretase inhibitors (GSI) led to prolonged survival in mouse models *in vivo*^[39]^. Since GSIs have toxic side effects in normal cells that express Notch, other ways of targeting Notch function^[70]^ could be considered as a useful strategy either alone or in combination with other drugs for FGFR1-driven T-lymphomas.

Activation of chimeric kinases also leads to activation of SRC^[71]^ and treatment of MLN-eo FGFR1 cells with the SRC inhibitor dasatinib led to reduced proliferation *in vitro* and prolonged survival *in vivo*. In a CNTRL-FGFR1 model of MLN-eo FGFR1, upregulation of MYC was detected and when the JQ1 MYC inhibitor was combined with ponatinib, there was a synergistic effect on suppression of leukemogenesis in human cell models propagated in immunocompromised mice^[38]^. Up regulation of MYC is one of the most consistent observations in MLN-eo FGFR1 cells, regardless of the chimeric kinase involved, since it is directly activated by FGFR1^[72]^. Using the 10054-F8 MYC inhibitor, cell proliferation was suppressed *in vitro* and this effect synergized with BJG398. One of the consequences of MYC activation is the upregulation of the MYB oncogene and treatment of MLN-eo FGFR1 cells with mebendazole leding to increased apoptosis and reduced cell viability as well as suppression of leukemogenesis *in vivo*^[73]^. Thus, a detailed analysis of genetic mechanisms of MLN-eo FGFR1 development have suggested other targets that might be effective in treatment of FGFR1-driven malignancies resistant to FGFR1 inhibitors.

BCR-FGFR1 is unique amongst the various chimeric kinases in that it has dual kinase activity, with a serine-threonine kinase (STK) provided by the BCR component of the chimeric kinase, which activates SHP2 kinase amongst other targets^[27]^. Targeting SHP2 with the SHP099 pharmacological agent^[74,75]^, which stabilizes SHP2 in an inactive conformation, reduced cell proliferation *in vitro* and leukemogenesis *in vivo* and, when combined with the BGJ398 FGFR1 inhibitor, showed synergistic effects *in vivo*^[27]^. When other MLN-eo FGFR1 cells expressing other chimeric kinases were treated with SHP099, the effects were less dramatic since they do not have STK activity. FGFR1 activation in MLN-eo FGFR1 is also accompanied by Rac activation^[76]^ and when MLN-eo FGFR1 T- and B- lymphomas were treated with the Ehop016 pan-RAC inhibitor^[77]^, cell viability *in vitro* and leukemogenesis of the BCR-FGFR-driven B-lymphomas *in vivo*, was also suppressed^[27]^. These studies begin to address the issue of identifying molecular targets that are specific to individual rearrangement-defined MLN-eo FGFR1 as part of a customized therapy approach.

Thus, while not directly overcoming the mechanisms of resistance to FGFR1 inhibitors, the targets identified through a fine-detail dissection of genetic events associated with lymphoma development in MLN-eo FGFR1 has provided alternative strategies that might be used in combination with FGFR1 inhibitors to prevent/overcome resistance to mono-therapies as well as provide alternative approaches to treat resistant cells. Importantly, a more detailed molecular analysis of tumors in individual MLN-eo FGFR1 patients at the time of diagnosis is required to be able to customize an effective treatment regimen for each patient. This will be particularly challenging given the rare nature of the disease, which precludes large clinical trials to evaluate the efficacy of individual approaches but, given the poor overall outcome, may require bold decisions to be made in the clinical management of these patients.

## CONCLUSION

Developing methods of overcoming resistance to therapies targeting the driver event in rare forms of cancer can be challenging, since there are insufficient patients to conduct extensive clinical trials and in a reasonable time to benefit the patients that develop resistance. The development of representative mouse models for leukemia and lymphomas, however, can be a valuable addition to the understanding of the basis of drug resistance and also provide insight into therapeutic strategies that might be alternatives when resistance arises when used in concert with a detailed molecular profiling of the primary disease. This is especially the case where the human disease is rare and opportunities to develop approaches to overcome resistance are limited. Pre knowledge of the tumor specific genetic events that may be responsible for resistance, therefore, can streamline decision making. In the case of FGFR1 driven neoplasms, mechanisms of resistance that have been suggested in animal studies can be monitored during the course of standard ant-FGFR1 treatment to possibly detect emergence of resistant clones, although it cannot be excluded that novel mechanisms may also arise. In either event, the availability of preclinical data suggesting alternative therapeutic strategies may streamline the decision process in selecting alternative approaches.
